# Scanning optoacoustic angiography for assessing structural and functional alterations in superficial vasculature of patients with post-thrombotic syndrome: A pilot study

**DOI:** 10.1016/j.pacs.2024.100616

**Published:** 2024-05-07

**Authors:** Svetlana Nemirova, Anna Orlova, Alexey Kurnikov, Yulia Litvinova, Viacheslav Kazakov, Irina Ayvazyan, Yu-Hang Liu, Daniel Razansky, Pavel Subochev

**Affiliations:** a Privolzhsky Research Medical University, 10/1 Minin & Pozharsky sq., Nizhny Novgorod 603950, Russia; b A.V. Gaponov-Grekhov Institute of Applied Physics, Russian Academy of Sciences, 46 Ulyanov Str., Nizhny Novgorod 603950, Russia; c Institute of Pharmacology and Toxicology and Institute for Biomedical Engineering and, Faculty of Medicine, University of Zurich, Winterthurerstrasse 190, Zurich 8057, Switzerland; d Institute for Biomedical Engineering, Department of Information Technology and Electrical Engineering, ETH Zurich, Wolfgang-Pauli-Strasse 27, Zurich 8093, Switzerland

**Keywords:** Scanning optoacoustic angiography, Post-thrombotic syndrome, Superficial vessels, Functional tests

## Abstract

This study highlights the potential of scanning optoacoustic angiography (OA) in identifying alterations of superficial vasculature in patients with post-thrombotic syndrome (PTS) of the foot, a venous stress disorder associated with significant morbidity developing from long-term effects of deep venous thrombosis. The traditional angiography methods available in the clinics are not capable of reliably assessing the state of peripheral veins that provide blood outflow from the skin, a key hallmark of personalized risks of PTS formation after venous thrombosis. Our findings indicate that OA can detect an increase in blood volume, diameter, and tortuosity of superficial blood vessels. The inability to spatially separate vascular plexuses of the dermis and subcutaneous adipose tissue serves as a crucial criterion for distinguishing PTS from normal vasculature. Furthermore, our study demonstrates the ability of scanning optoacoustic angiography to detect blood filling decrease in an elevated limb position versus increase in a lowered position.

## 1 Introduction

Thrombosis occurring in various locations is of interest to physicians across all specialties, as its effects and consequences can impact all organs and systems. Acute arterial thrombosis leading to heart attacks and strokes remains a leading cause of death, while thrombosis in the lower extremities due to obliterating atherosclerosis and diabetic angiopathy comprises a significant cause of disability. Acute disruption of mesenteric circulation may require emergency surgery, placental thrombosis may lead to miscarriage, and thrombosis of the retinal arteries may result in blindness [1], [2], [3]. With venous thrombosis, patients may develop dysfunctions in any affected organs, as well as embolic complications such as pulmonary artery occlusion, which can lead to instant death or gradually developing changes that significantly reduce the quality of life. Due to the novel coronavirus infection pandemic, there has been an increase in the number of patients with chronic post-thrombotic changes, as evidenced by a pool of publications on COVID-associated thrombosis in the literature [4], [5].

The most frequently described post-thrombotic changes occur in the veins of lower extremities. These changes begin from the moment of stenosis or occlusion of the thrombosed vessel and can persist and even worsen throughout the patient's life. Factors that predispose to the development of post-thrombotic disease include damage to the iliac veins, increased body weight of the patient, severity and recurrence of the disease [6], [7], which often occurs with the remaining components of the Virchow’s triad, including slowing blood flow with phlebohypertension, as well as trauma to the vascular machine with long-term inflammation. The formation of post-thrombotic syndrome (PTS) is also associated with the development of venous trophic ulcers, which are more likely to occur in patients with initial disorders of tissue trophism such as diabetes mellitus and varicose veins [8]. An assessment of the state of peripheral vessels that provide blood outflow from the skin could enable an objective evaluation of personalized risks of PTS formation after venous thrombosis. Various methods are available for non-invasive assessment of peripheral vessel morphology and presence of blood flow, such as laser Doppler flowmetry, photoplethysmography, computer-assisted venous occlusion plethysmography, as well as capillaroscopy and optical coherence tomography (OCT) [9], [10], [11]. However, these methods do not allow the assessment of larger "intermediate" veins that are not accessible to traditional ultrasound. Currently, vessels of this caliber have largely been overlooked due to the absence of a non-invasive method that allows for longitudinal studies.

Optoacoustic (OA) imaging is a promising approach for obtaining information about the distribution of subcutaneous vessels [12]. As compared to optical imaging methods, OA allows deeper penetration into biological tissues (up to several millimeters) while retaining high spatial resolution in the several tens of microns range. The images are formed by detecting and reconstructing ultrasonic waves generated via absorption of short laser pulses by tissue chromophores, primarily hemoglobin. Scanning optoacoustic mesoscopy systems are well suited for dermatological applications and imaging of superficial vasculature in humans [13]. To obtain a three-dimensional image, divergent radiation is delivered to the tissue through an optical fiber and a raster scan of a focused broadband detector is performed.

Currently, OA is utilized to provide functional and anatomical characteristics of various vasculopathies. For instance, in patients with Raynaud's syndrome, a reduction in blood oxygen saturation levels has been observed when compared to healthy volunteers [14]. Peripheral artery disease patients were found to have low muscle hemoglobin content and oxygenation [15]. Multispectral optoacoustic tomography has proven promising for staging peripheral arterial disease based on muscle oxyhemoglobin concentrations [16]. Differences in dermal vessel density were demonstrated in normal conditions and in port wine stains [17].

Various functional tests based on controlled effect on tissue perfusion are utilized to assess the vascular system condition in different pathologies [18]. Among them changes in limb position are based on decrease in inflow and increase in outflow of blood from the studied organ.

In this work, using raster-scan-based OA mesoscopy we demonstrate, for the first time, the structural and functional alterations in superficial vasculature during repositioning of a healthy limb and in post-thrombotic syndrome.

## 2 Materials and methods

The main group included four patients (51–66 years old) with anamnestic and clinically identified signs of PTS, as confirmed by ultrasound. In the 2–3 years period prior to the present study, all patients with PTS had suffered occlusive thrombosis of the femoral vein, confirmed clinically and by ultrasound. In all cases, the patients had been treated with oral anticoagulants in a therapeutic dose for up to 6 months. After one year, all consulted a surgeon with complaints of swelling and periodic pain in the lower extremities. Directly upon inclusion in the experimental group, all patients were interviewed and examined to identify characteristic complaints: in all cases, patients reported daily swelling of the lower extremities and pain, which developed during the day with maximum severity in the evening, without visually detectable varicose transformation of the saphenous veins. Ultrasound examination revealed signs of previous thrombosis in the form of thickening of the venous wall, the presence of intraluminal hyperechoic cord-like or ribbon-like structures, partially blocking the lumen of the vein, and fragmentarily creating multichannel blood flow, fixing deformed valve leaflets with signs of their insufficiency. Exclusion criteria from the study were the presence of skin diseases and hyperpigmentation of the skin in the studied area, anemia, as well as edema and vascular diseases of other etiologies. The control group consisted of four volunteer (21–39 years old) without vascular pathology. Adult men and women who did not have a history of complaints similar to those associated with venous insufficiency of any etiology and who did not have visually or palpably detectable signs of diseases affecting the veins, arteries, and lymphatic vessels at the time of participation in the experiment, were included into the study. They had no history of taking medications or undergoing surgical interventions for vascular diseases, nor pathological ultrasound findings in the arteries and veins of the lower extremities. All experiments were approved by the Ethical Committee of Privolzhsky Research Medical University (Protocol #7 from 06.05.2022).

**Fig. 1:**
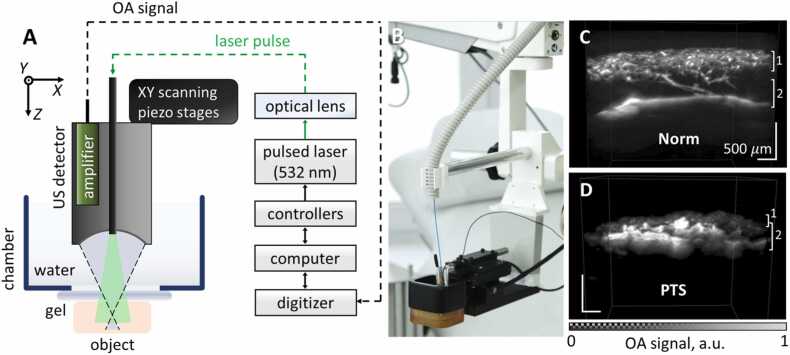
Scanning optoacoustic angiography of the human foot. (A) Scheme of the experimental setup. (B) Photograph of the experimental setup. (C) Cross-sectional OA images of healthy foot vessels. (D) The corresponding images recorded from a patient with PTS. 1 - vascular plexuses of the dermis, 2 - vessels of subcutaneous adipose tissue.

The scanning OA mesoscopy system, whose detailed description is available elsewhere [19], consisted of two scanning stages (25 × 25 mm^2^ range; 25 μm step size), a pulsed laser source (532 nm wavelength; 1 ns pulse duration; 2 kHz repetition rate) and a digitizer (16 bit vertical resolution; 200 MHz sampling rate). A bright-field OA scanner based a customized spherical wideband (1–50 MHz at 10 dB level) polyvinylidene difluoride detector (BARI-NN Ltd., Russia), integrated with 400 μm diameter multimode optical fiber, was employed for imaging (Figs. 1A,B), providing <50 μm spatial resolution in the lateral dimensions [20].

Examination of all patients was carried out under the same conditions: before the start of scanning, the patient stayed put in a supine position for 10 minutes, then for 10 minutes in each of the following positions: (1) horizontal arrangement of the examined limb; (2) the limb is oriented upward at 30° angle relative to the horizontal plane of the heart; (3) the limb is pointing down at 30° angle relative to the horizontal plane of the heart. For the study, a region of interest was selected 2 cm distal to the medial malleolus, in an area of the most significant effect of phlebohypertension on tissue trophism and the most frequent formation of trophic ulcers of venous etiology. Prior to scanning, a replaceable immersion chamber filled with water was attached under the OA sensor. An immersion OA detector was brought into acoustic contact with the skin through a thin layer of medium-viscosity ultrasonic gel Mediagel (Geltek-Medica LTD, Russia) and transparent film (less than 100 µm thick) both located at the bottom of water-filled immersion chamber. Tissue pressing by the immersion chamber was controlled using a Z-stage built into the OA system. To align ∼2 mm visualization depth provided by the laser radiation wavelength (532 nm) with ∼ 2 mm depth of the field of OA detector, the scanning plane had to be brought in parallel with non-planar skin surface. For convenient lateral alignment, we started each investigation with preliminary volumetric OA imaging (10 × 10 mm^2^ area with 100 µm step), and proceeded by selection of a smaller area for final scanning (5 × 5 mm^2^, 50 µm step). Final scanning required less than 30 seconds, but required for the human to stay in still position to obey motion artefacts.

To improve the signal to noise ratio of blood vessels in OA data, a set of two digital filters was applied to each raw A-scan [21]. Unwanted high-frequency noise and signals from structures smaller than the scanning step size were eliminated by 50 MHz low-pass filter. Low frequency offset signals from the bulk tissues were further suppressed by 0.2 MHz high-pass filter. The filtered 3D datasets were then processed by delay-and-sum reconstruction in Fourier domain [22]. To quantify the parameters of the vascular bed observed in the OA images (blood volume, vessel diameter and tortuosity), numerical data processing methods were applied to the reconstructed datasets. In particular, blood volume was estimated in the 3D data domain using Avizo (Thermo Scientific, USA). First, all the blood vessels observed in the reconstructed 3D images obtained in a horizontally positioned limb were segmented. 3D segmentation was processed by manually adjusting binarization threshold value for each imaged individual. Same pre-adjusted individual threshold values were further used to binarize blood vessels observed in all the corresponding upward/horizontal/downward limb positions. The values of blood volume were determined for each position, as a percentage of the volume occupied by binarized vessel structures in relation to the total image volume.

The diameters and tortuosity of the blood vessels were estimated in the image domain. The OA images corresponding to each individual were obtained as maximum intensity projections of the reconstructed 3D datasets along the Z direction. Then, contrast enhancement and quadratic interpolation smoothing filters were applied to the images [23]. Binarization and skeletonization of individual blood vessels was provided by automatic adjustment of threshold values using Otsu method [24]. The vessel diameter was calculated using a custom PostProGUI Matlab program with a graphical user interface, which allowed for a manual labeling of the individual images by marking the vessel contours to indicate their key rotation points. The vessel tortuosity was then calculated as the relative difference between the length of the labeled vessels and the length of the labeled chords [25].

All measured values were plotted as means ± SD. For statistical analysis, the IBM SPSS Statistics software was used. T-test for independent samples was performed to estimate the significance of the differences between normal and pathologic tissues. One-way ANOVA with Bonferroni correction was performed to estimate significance of the differences between the limb positions. Statistically significant value was taken as p < 0.05. The Pearson correlation coefficient was calculated to assess the correlation between the values of vessel tortuosity and vessel diameter.

## 3 Results

Exemplary OA images depicting dermal and subcutaneous blood vessels in the foot of a healthy volunteer versus a patient with post-thrombotic syndrome are shown in Figs. 1C and 1D, respectively. Areas with elevated OA signal levels highlight the hemoglobin-containing vessels. Superficially located vessels of the plexuses in the dermis (at up to 1 millimeter depth) have a smaller diameter as compared to underlying vessels in the subcutaneous adipose tissue with up to 300 microns diameter. In normal tissues, subcutaneous adipose tissue vessels and vascular plexuses of the dermis are clearly distinguishable. In PTS, veins of the subcutaneous tissue are sharply dilated and tortuous with the vascular layers overlapping each another and being indistinguishable.

**Fig. 2:**
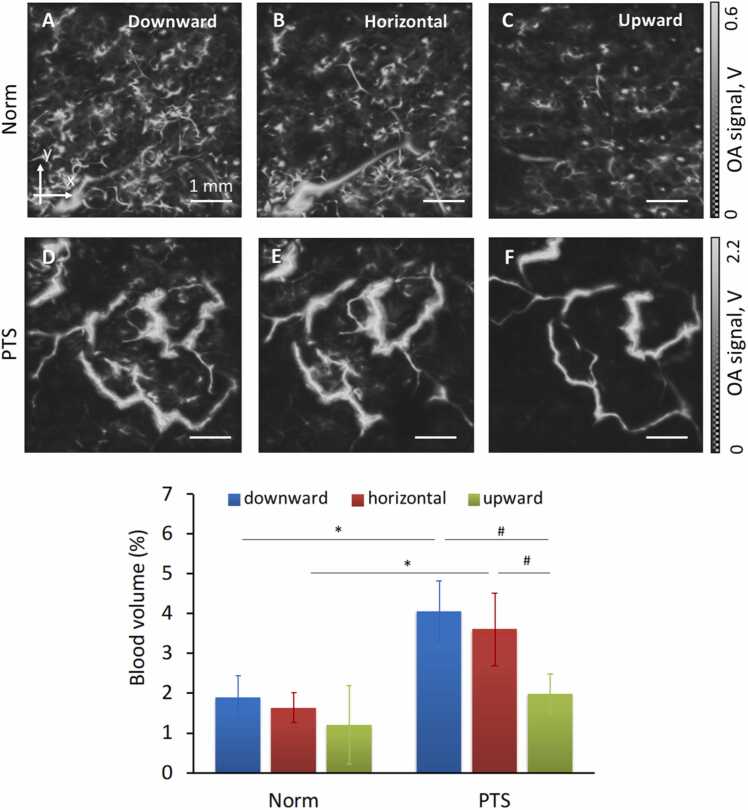
OA images of the dermal and subcutaneous blood vessels in the foot of healthy volunteers (A-C) versus patients with PTS (D-F). Three limb orientations were considered, namely, downward (A, D), horizontal (B, E) and upward (C, F). The blood volume values calculated from the OA images are shown in (G). ∗ - statistically significant differences between the healthy and PTS groups; # - statistically significant differences between orientations of the extremities.

Fig. 2 demonstrates en-face OA images of healthy and PTS vessels of the differently oriented foot. The images of a healthy foot are mostly manifested with small vessels of the dermis with the large deeper located vessels of the subcutaneous adipose tissue being less distinguishable. In a healthy limb elevation causes a decrease in the OA signal associated with the outflow of blood from both small (superficial) and large (deep) vessels (Fig. 2C). Lowering the foot causes an increase in blood supply in the small vessels, which results in a clear manifestation of the superficially located network of small and dense vessels (Fig. 2A). Total blood volume in healthy tissues was not altered significantly by changing the limb position (Fig. 2G). Images of the PTS tissues demonstrate a decrease in the number of small superficial vessels, while subcutaneous large vessels are visualized more clearly. Position-dependent changes are also manifested as an increase of OA signal in the downward limb orientation and a signal decrease in the upward orientation (Fig. 2D-F). Tissue blood volume in patients with PTS changes significantly with change of the extremity position (p < 0.05) (Fig. 2G). At the same time, blood volume significantly increased (p < 0.05) in PTS patients as compared to the normal values for almost all orientations.

**Fig. 3:**
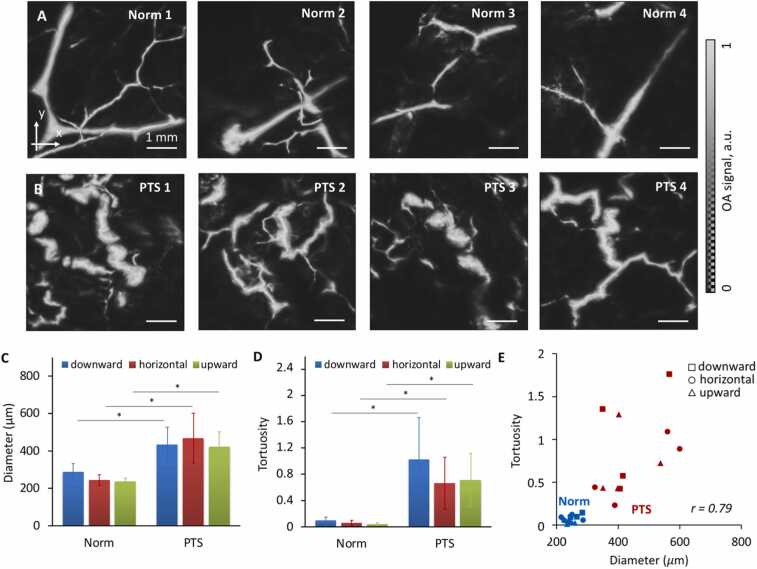
OA images of blood vessels of subcutaneous adipose tissue of foot in healthy volunteers (A) and patients with PTS (B) in a horizontal limb orientation. Vessel diameter (C) and tortuosity (D) calculated from the OA images in the downward, horizontal and upward limb orientation. Vessel tortuosity values versus vessel diameter values are shown in (E). ∗ - statistically significant differences between healthy and PTS groups.

Fig. 3 demonstrates the vessels of subcutaneous adipose tissue of the foot in all the healthy volunteers and PTS patients after manual removal of a layer of small superficial vessels from the OA images. As can be seen from the OA images, healthy tissues are characterized by a regular arrangement of almost straight non-dilated blood vessels (Fig. 3A). Compared with the normal vasculature, the vessels in PTS were found to be curved and significantly dilated (Fig. 3B) with their average diameter increased by a factor of two (p < 0.05). Neither vessel diameter nor tortuosity depended on the limb orientation (Fig. 3C,D).

Slight fluctuations in the vessel tortuosity occurred when changing the limb orientation in the control group with the changes being minimal in the elevated limb orientation and more significant when the limb is lowered (Fig. 3D). When examining patients with PTS, attention is drawn to the increase in the vessel tortuosity, which increases in the downward orientation. The degree of vessel tortuosity in PTS exceeded that in normal tissues by approximately 13 times (p < 0.05). An increase in the degree of vessel tortuosity may be associated with the rise of their diameter. A strong positive correlation (r = 0.79, p < 0.01) was revealed between these parameters (Fig. 3E).

## 4 Discussion

Post-thrombotic disease is associated with pathological restructuring of vessels of different calibers. In our work, we used OA to compare the vessels of plexuses in the dermis and subcutaneous adipose tissue of a healthy foot to those of patients with PTS and proposed criteria for diagnosis and follow-up.

The first clinical sign noted in patients with PTS is a violation of the angioarchitectonics in the dermal and subcutaneous adipose tissue layers [26] whose vasculature becomes indiscernible (Fig. 1, Fig. 2). In the horizontal foot position, patients with PTS have a relatively more superficial arrangement of larger vessels that compress the peripheral plexuses.

The second parameter is an increase in the vessel diameter in the subcutaneous tissues of the foot in PTS patients (Fig. 2). Vasodilatation is associated with diminished blood drainage through the main deep veins, which is characteristic of patients with PTS due to partial occlusion of the lumen by organized thrombotic masses and multidirectional flow due to valvular insufficiency and vertical reflux. The thin muscular layer of the peripheral veins does not compensate for the effect of high phlebohypertension [27], the vascular wall appears stretched and the diameter of the lumen increases.

The third parameter detected by OA, which is particularly promising for dynamic observations, was the blood volume. In PTS, against the background of severe phlebohypertension, when lowering the limb, the total volume of the vessels increases not only due to an increase in diameters but also due to the greater tortuosity of the veins. In patients with PTS, there was no significant decrease in the overall density of hemoglobin-containing structures during limb elevation, which indirectly indicates a permanent expansion of the veins in PTS corresponding to a pathological remodeling of the vascular wall.

OA further allows visualizing another key sign appearing in PTS, namely, the tortuosity of subdermal vessels (Fig. 2), which is similar to that described in capillaroscopy in patients with chronic venous insufficiency [28]. This shows that the formation of secondary varicose veins accompanying the PTS development [29] may in fact originate from the subdermal vascular bed. Our OA study revealed that repositioning a healthy limb leads to fluctuations in the vessel blood supply, i.e. the blood volume increases when the limb is lowered and vice versa when it is elevated (Fig. 2).

Changes in diameter of the main vein depending on the limb orientation have previously been described [30]. Our OA study revealed further alterations in the vessels of the dermal and subdermal plexuses, which can be attributed to the pressure increase of the liquid column and expansion of the lumen for the downward limb orientation. Conversely, a decrease in the blood inflow and, accordingly, blood pressure occurs for the upward orientation. Our study clearly shows the effect of an increase in the inner diameter of a vessel (accumulation of hemoglobin in its lumen) on its tortuosity (Fig. 3E). The contour of the inner wall appears stretched in all direction, arguably due to decompensated phlebohypertension.

From a clinical perspective, OA offers several advantages for imaging microcirculatory changes in patients with PTS. In standard ultrasound examinations for PTS, vessels that are located at a depth of 5 mm or less are visible. However, at a depth of 2 mm, it is not possible to differentiate the branching of peripheral vessels due to the limited resolution of the method. In contrast, OA can visualize peripheral vessels with several levels of branching at this depth, thereby complementing the data obtained from ultrasound. This is particularly valuable because it involves the vessels that drain peripheral tissues where trophic disorders may develop during PTS.

In our study, we demonstrated the capabilities of OA imaging for non-invasive observation of changes in intermediate-caliber vessels, which were previously only accessible through ex vivo methods. Moreover, during ultrasound examinations of patients in PTS group (data not shown), no significant pathological enlargement of the subcutaneous veins was observed, which excluded secondary varicose transformation of the vessels and may lead to an underestimation of the severity of pathological changes. However, the pronounced dilation and tortuosity of the peripheral veins, revealed by OA, provided evidence of early pathological remodeling of the peripheral vessels. Confirming these findings in a larger sample could aid in the early diagnosis of disease progression and help exclude edema of other etiologies.

Superficial small vessels are the primary factor influencing OA signal alterations. In PTS, the signal from the proximal vessels is more pronounced, which is associated with their greater blood supply and a more superficial location of the subdermal veins due to their expansion and compression of the distal vessels of the dermal plexuses.

Importantly, the identified features of the peripheral vascular bed differed from the previously reported age-related changes and were specific for the group of patients with PTS. A gradual decrease in the density and diameter of healthy human skin vessels with age has been demonstrated in studies by other groups [31], [32] and in our previous work [33]. However, the current study reveals an increase in blood volume and vessel diameter in PTS, suggesting that these changes are independent of age-related effects.

## 5 Conclusions

Our pilot study demonstrated the possibilities of non-invasive OA imaging in differentiating vascular features of the dermal and subdermal vascular plexuses in normal and PTS patients during positional tests. The observations open up new prospects for real-time monitoring of the effectiveness of drugs with angioprotective properties and other methods of prevention and treatment of patients with PTS. Moreover, an important area of application for this method may be the differential diagnosis between PTS, angiopathies and other diseases.

## Funding

The development of optoacoustic system, clinical experiments and data processing were supported by 10.13039/100000935RSF project 19–75–10055. The development of an algorithm for tomographic optoacoustic reconstruction was supported by the Center of Excellence "Center of Photonics" funded by the Ministry of Science and Higher Education of the Russian Federation, Contract No. 075–15–2022–316. The development of optical focusing system was supported in the frames of the Governmental Project of the Institute of Applied Physics RAS (Project #FFUF-2021–0014). DR acknowledges support from the Swiss Cancer Research under grant KFS-5234–02–2021.

## CRediT authorship contribution statement

**Alexey Kurnikov:** Writing – original draft, Methodology, Investigation. **Yulia Litvinova:** Visualization, Investigation, Data curation. **Svetlana Nemirova:** Writing – original draft, Methodology, Investigation, Conceptualization. **Anna Orlova:** Writing – review & editing, Writing – original draft, Formal analysis. **Yu-Hang Liu:** Writing – original draft, Visualization, Methodology. **Daniel Razansky:** Writing – review & editing, Writing – original draft, Supervision, Resources. **Viacheslav Kazakov:** Writing – original draft, Investigation. **Irina Ayvazyan:** Writing – original draft. **Pavel Subochev:** Writing – review & editing, Writing – original draft, Supervision, Funding acquisition, Conceptualization.

## Declaration of Competing Interest

The authors declare the following financial interests/personal relationships which may be considered as potential competing interests: Pavel Subochev reports financial support was provided by Russian Science Foundation. If there are other authors, they declare that they have no known competing financial interests or personal relationships that could have appeared to influence the work reported in this paper.

## Data Availability

Data will be made available on request.

## References

[1] Gnanapandithan K., Feuerstadt P. (2020). Mesenteric Ischemia. Curr. Gastroenterol. Rep..

[2] Middeldorp S., Naue C., Köhler C. (2022). Thrombophilia, thrombosis and thromboprophylaxis in pregnancy: for what and in whom?. Hamostaseologie.

[3] Schwaber E.J., Fogelman N., Sobol E.K., Mehrotra D., Powell J.A., Mian U., Gritz D.C. (2018). Associations with retinal vascular occlusions in a diverse, urban population. Ophthalmic Epidemiol..

[4] Paul L., Jain T., Singh S. (2022). Retinal manifestations of ophthalmic artery occlusion with ischemic stroke in a young patient with COVID-19. Indian J. Ophthalmol..

[5] Cheng N.M., Chan Y.C., Cheng S.W. (2022). COVID-19 related thrombosis: a mini-review. Phlebology.

[6] Rabinovich A., Ducruet T., Kahn S.R. (2018). Development of a clinical prediction model for the postthrombotic syndrome in a prospective cohort of patients with proximal deep vein thrombosis. J. Thromb. Haemost..

[7] Cucuruz B., Kopp R., Pfister K., Noppeney J., Tripal K., Korff T., Zeman F., Koller M., Noppeney T. (2020). Risk and protective factors for post-thrombotic syndrome after deep venous thrombosis. J. Vasc. Surg. Venous Lymphat. Disord..

[8] Galanaud J.-P., Bertoletti L., Amitrano M., Fernández-Capitán C., Pedrajas J.M., Rosa V., Barrón M., Lorenzo A., Madridano O., Quéré I., Kahn S.R., Prandoni P., Monreal M. (2018). Predictors of post-thrombotic ulcer after acute DVT: the RIETE registry. Thromb. Haemost..

[9] El Miedany Y., Ismail S., Wadie M., Hassan M. (2022). Nailfold capillaroscopy: tips and challenges. Clin. Rheumatol..

[10] Christ F. (1996). Methods of microcirculatory monitoring (laser Doppler flowmetry, photoplethysmography and computer-assisted venous occlusion plethysmography). Anasthesiol Intensiv. Notf. Schmerz.

[11] Petrova K.S., Nemirova S.V., Petrova G.A., Karpenko A.A., Ryabkov M.G. (2022). Three-dimensional optical coherence tomography: possibilities in assessing the microvasculature of the skin. Proc. SPIE 11934, Photonics Dermatol. Plast. Surg..

[12] Omar M., Aguirre J., Ntziachristos V. (2019). Optoacoustic mesoscopy for biomedicine. Nat. Biomed. Eng..

[13] Dean-Ben X.L., Razansky D. (2021). Optoacoustic imaging of the skin. Exp. Dermatol..

[14] Eisenbrey J.R., Stanczak M., Forsberg F., Mendoza-Ballesteros F.A., Lyshchik A. (2018). Photoacoustic, Oxygenation quantification in patients with Raynaud's: first-in-human results. Ultrasound Med. Biol..

[15] Karlas A., Masthoff M., Kallmayer M., Helfen A., Bariotakis M., Fasoula N.A., Schäfers M., Seidensticker M., Eckstein H.H., Ntziachristos V., Wildgruber M. (2021). Multispectral optoacoustic tomography of peripheral arterial disease based on muscle hemoglobin gradients-a pilot clinical study. Ann. Transl. Med..

[16] Günther J.S., Knieling F., Träger A.P., Lang W., Meyer A., Regensburger A.P., Wagner A.L., Trollmann R., Woelfle J., Klett D., Uter W., Uder M., Neurath M.F., Waldner M.J., Rother U. (2023). Targeting muscular hemoglobin content for classification of peripheral arterial disease by noninvasive multispectral optoacoustic tomography. JACC Cardiovasc Imaging.

[17] Ma H., Cheng Z., Wang Z., Qiu H., Shen T., Xing D., Gu Y., Yang S. (2021). Quantitative and anatomical imaging of dermal angiopathy by noninvasive photoacoustic microscopic biopsy. Biomed. Opt. Express.

[18] Wright C.I., Kroner C.I., Draijer R. (2006). Non-invasive methods and stimuli for evaluating the skin's microcirculation. J. Pharmacol. Toxicol. Methods.

[19] Subochev P. (2016). Cost-effective imaging of optoacoustic pressure, ultrasonic scattering, and optical diffuse reflectance with improved resolution and speed. Opt. Lett..

[20] Li W., Hofmann U.A., Rebling J., Zhou Q., Chen Z., Ozbek A., Gong Y., Subochev P., Razansky D., Dean-Ben X.L. (2022). Broadband model-dased optoacoustic mesoscopy enables deep-tissue imaging beyond the acoustic diffraction limit. Laser Photonics Rev..

[21] Treeby B.E., Cox B.T. (2010). k-Wave: MATLAB toolbox for the simulation and reconstruction of photoacoustic wave fields. J. Biomed. Opt..

[22] Subochev P., Spadin F., Perekatova V., Khilov A., Kovalchuk A., Pavlova K., Kurnikov A., Frenz M., Jaeger M. (2021). Toward real-time giga-voxel optoacoustic/photoacoustic microscopy: GPU-accelerated fourier reconstruction with Quasi-3D implementation. Photonics.

[23] Rebling J., Ben-Yehuda Greenwald M., Wietecha M., Werner S., Razansky D. (2021). Long-term imaging of wound angiogenesis with large scale optoacoustic microscopy. Adv. Sci..

[24] Liu Y.H., Brunner L.M., Rebling J., Ben-Yehuda Greenwald M., Werner S., Detmar M., Razansky D. (2022). Non-invasive longitudinal imaging of VEGF-induced microvascular alterations in skin wounds. Theranostics.

[25] Ramos L., Novo J., Rouco J., Romeo S., Alvarez M.D., Ortega M. (2018). Retinal vascular tortuosity assessment: inter-intra expert analysis and correlation with computational measurements. BMC Med. Res. Methodol..

[26] Imanishi N., Kishi K., Chang H., Nakajima H., Aiso S. (2008). Three-dimensional venous anatomy of the dermis observed using stereography. J. Anat..

[27] Cockett F.B. (1954). Abnormalities of the deep veins of the leg. Postgrad. Med. J..

[28] Petrov S.V., Azizov G.A., Kozlov V.I. (2007). Microcirculation disorders in chronic venous insufficiency of the lower extremities and its assessment by non-invasive research methods. Fundam. Res..

[29] Meissner M.H., Eklof B., Smith P.C., Dalsing M.C., DePalma R.G., Gloviczki P., Moneta G., Neglén P., Donnell T.O.', Partsch H., Raju S. (2007). Secondary chronic venous disorders. J. Vasc. Surg..

[30] Czyzewska D., Ustymowicz A., Kowalewski R., Zurada A., Krejza J. (2017). Cross-sectional area of the femoral vein varies with leg position and distance from the inguinal ligament. PLoS One.

[31] Gunin A., Petrov V., Vasilieva O., Golubtsova N. (2015). Age-related changes of blood vessels in the human dermis. Adv. Gerontol..

[32] Hara Y., Yamashita T., Kikuchi K., Kubo Y., Katagiri C., Kajiya K., Saeki S. (2018). Visualization of age-related vascular alterations in facial skin using optical coherence tomography-based angiography. J. Dermatol. Sci..

[33] Perekatova V., Kirillin M., Nemirova S., Orlova A., Kurnikov A., Khilov A., Pavlova K., Kazakov V., Vildanov V., Turchin I., Subochev P. (2022). Quantitative characterization of age-related changes in peripheral vessels of a human palm using raster-scan optoacoustic angiography. Photonics.

